# Repositioning of acefylline as anti-cancer drug: Synthesis, anticancer and computational studies of azomethines derived from acefylline tethered 4-amino-3-mercapto-1,2,4-triazole

**DOI:** 10.1371/journal.pone.0278027

**Published:** 2022-12-15

**Authors:** Irum Shahzadi, Ameer Fawad Zahoor, Burak Tüzün, Asim Mansha, Muhammad Naveed Anjum, Azhar Rasul, Ali Irfan, Katarzyna Kotwica-Mojzych, Mariusz Mojzych

**Affiliations:** 1 Department of Chemistry, Government College University Faisalabad, Faisalabad, Pakistan; 2 Plant and Animal Production Department, Technical Sciences Vocational School of Sivas, Sivas Cumhuriyet University, Sivas, Turkey; 3 Department of Applied Chemistry, Government College University Faisalabad, Faisalabad, Pakistan; 4 Department of Zoology, Government College University Faisalabad, Faisalabad, Pakistan; 5 Laboratory of Experimental Cytology, Medical University of Lublin, Lublin, Poland; 6 Department of Chemistry, Siedlce University of Natural Sciences and Humanities, Siedlce, Poland; Vignan Pharmacy College, INDIA

## Abstract

Novel azomethines derived from acefylline tethered triazole hybrids (**7a-k**) have been synthesized and evaluated against human liver cancer cell line (Hep G2) using MTT assay. The synthesized series of azomethines exhibited promising efficacy against liver cancer cell line. Screening of the synthesized series identified compound **7d** with the least cell viability value (11.71 ± 0.39%) as the most potent anticancer agent in contrast to the reference drug acefylline (cell viability = 80 ± 3.87%). In this study, the potentials of the novel agents (**7a-k**) to inhibit liver cancer proteins were assessed. Subsequently, the structure-activity relationship of the potential drug candidates was assessed via ADME/T molecular screening. The cytotoxic potential of these derivatives was also investigated by hemolysis and thrombolysis. Their hemolytic and thrombolytic studies showed that all of these drugs had very low cytotoxicity and moderate clot lysis activity. Compound **7g** (0.26% hemolysis) and **7k** (52.1% clot lysis) were the least toxic and moderate thrombolytic agents respectively.

## Introduction

Azomethine structural cores are pervasive in a range of pharmaceutically active heterocyclic scaffolds with potent activities such as antibacterial [1], antifungal [2,3], antimalarial, antimycobacterial [4–6], antimicrobial [7], cytotoxic, anticonvulsant [8] antiproliferative, and anticancer agents [9,10]. Purine is considered to be an important heterocycle in life practices and libraries of purines are examined against a variety of biological targets. Theophylline is present in the class of purine-based xanthine alkaloids which is widely used to treat respiratory diseases [11]. Theophylline is also used to enhance blood pressure and relax bronchial smooth muscle. It has anti-inflammatory properties and has been found to be effective in controlling chronic obstructive pulmonary disease (COPD) [12]. It has been observed that hydrogen bonding can increase DNA binding affinity by complicating DNA with theophylline, which can also act as an antioxidant [13]. In addition, theophylline has been diagnosed as an adenosine antagonist to prevent contrast-induced nephropathy (CIN), which is associated with kidney failure and has been shown to be effective in preventing CIN [14]. Fortunately, some theophylline derivatives, such as theophylline nucleoside derivatives, are also effective against the hepatitis B virus [15]. Some nitrates of theophylline derivatives were found to have a strong analgesic effect in hypertensive mice [16].

A well-known derivative of theophylline is acefylline, also known as acetyloxy theophylline. Acefylline and its analogs are potent pharmacological molecules commonly known as bronchodilators for the treatment of lungs disorder such as acute asthma [17]. In addition to bronchodilation, many biological activities have been reported in acefylline derivatives such as anticancer [18], antituberculosis [19], and anti-asthmatic [20]. Moreover, it is also used as a cardiac stimulant, diuretic [21], and adenosine antagonist receptor [22]. Because of these properties, acefylline has become attractive for researchers.

**Fig 1:**
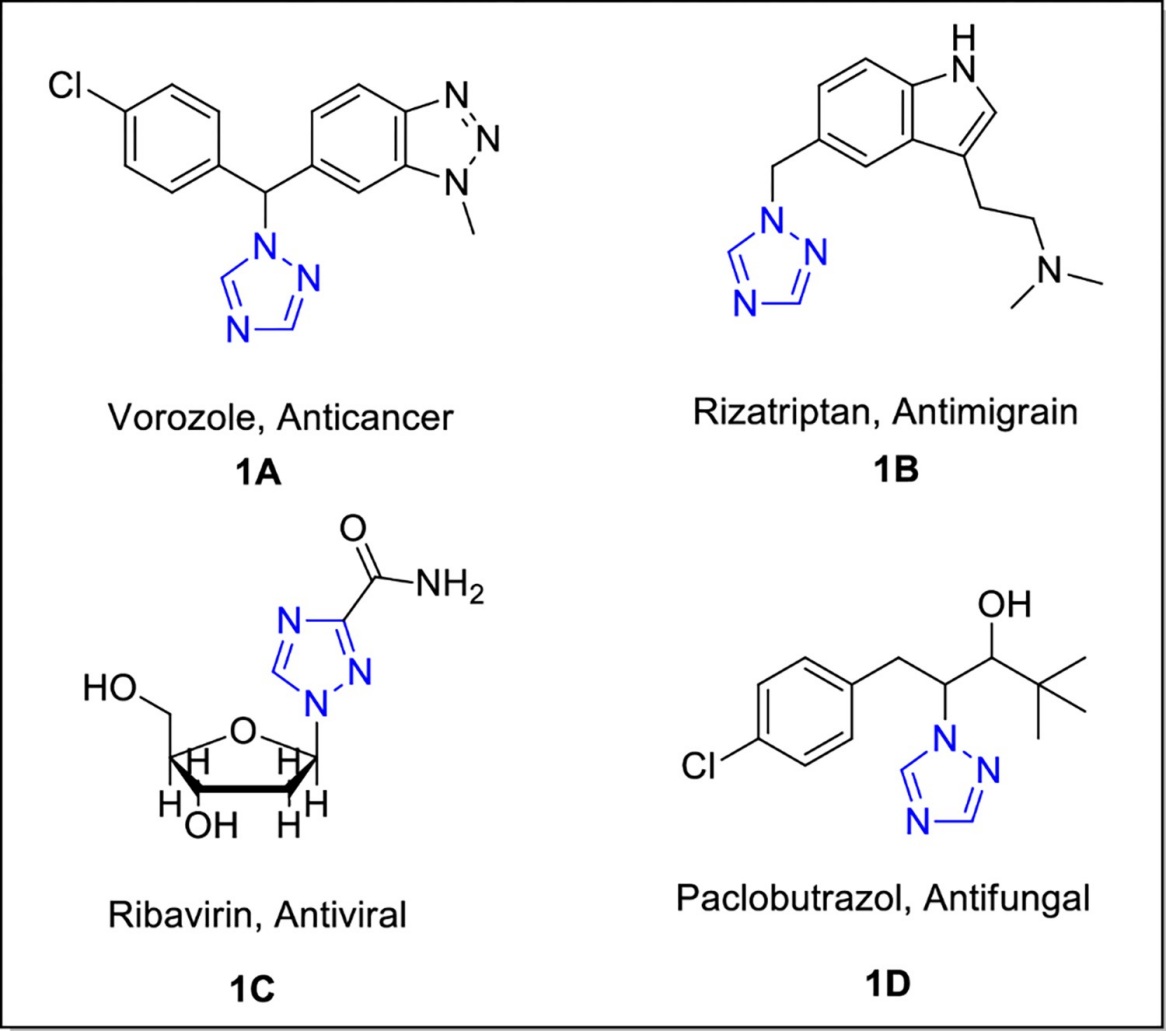
Bioactive triazole-based drugs: Vorozole (1A) Rizatriptan (1B), Ribavirin (1C) and Paclobutrazol (1D).

On the other hand, the 1,2,4-triazole core has been found to have significant biological activities like analgesic [23], local anesthetic, antimalarial [24], antimicrobial, antineoplastic [25], antiviral [26] anticonvulsant [27] and anti-cancer activities [28]. Various triazole-based compounds **(Fig 1)** have been discovered and are being used as medicine [29].

Our research group has already reported Schiff bases [30] 1,3,4-oxadiazoles [31] and 1,2,4-triazoles [32,33] substituted acefylline derivatives as anti-cancer agents. Molecular modeling studies enable the theoretical assessment and prediction of the activity of the drugs by optimization of the structural orientation of molecules with respect to the binding site. Theoretical calculations provide the convenience of making the activities of molecules against cancer proteins quickly and reliably before experimental procedures [34]. By examining high activity molecules with theoretical calculations, the synthesis of more active and more efficient molecules is possible [35]. In continuation of our work on the design and synthesis of novel candidates with potential therapeutic applications [36–38] herein, we report novel azomethine derived from acefylline-1,2,4-triazole hybrids and evaluate their antitumor activity against liver cancer HepG2 cells. To study the activities of molecules against liver cancer proteins, eliminated in liver cancer two protein (DLC2) (PDB ID:2H80) [35], by residual dipolar couplings (PDB ID:2JW2) [39] and crystal structure of the hepatocellular carcinoma-associated protein (PDB ID: 3WZE) [40] were used. Molecular docking studies followed by ADME/T analysis revealed the structure-activity relationship by prediction of binding modes of the title molecules with target proteins.

## Materials and methods

### Chemistry

The reagents and solvents used in this work were of Alfa Aesar, Merck, and Sigma Aldrich and used as it is. Anhydrous sodium sulfate was used to dry extracted organic layers. IR spectra (ν, cm^−1^) of synthesized series of compounds were obtained by Bruker FT-IR spectrometer using KBr pellets. NMR spectra were recorded on a Bruker spectrometer (400 MHz, model AV-400). The chemical shift values (δ) were measured in ppm using DMSO solvent. Melting points (m.p) of the synthesized derivatives were observed using Gallenkamp equipment. Pre-coated silica gel 60 F254 TLC was used to monitor reaction using analytical grade solvents like methanol, dichloromethane, ethyl acetate, and *n*-hexane.

#### Synthesis of 4-amino-1,2,4-triazole from acefylline-1,3,4-oxadiazole hybrids (6)

A solution of oxadiazole derivative of acefylline (0.2 g, 0.00067mol) was heated under reflux with hydrazine monohydrate (0.3 g, 0.0061mol) for 6 hours. The reaction completion was monitored using TLC. After reaction completion mixture was cooled overnight. To afford the amino triazole as product the reaction mixture was than filtered and recrystallized.

White powder; Yield: 70%; m.p 217°C; IR: υ 1648 (CO-xanthine); 1556 (C = C); 1544 (C = N); 1472 (CH_2_); 600–700 (C-S); 2500–2600 (S-H). 400 MHz (^1^H-NMR, DMSO-*d*_6_, δ/ppm): 3.18, 3.33 (s, 6H, 2NCH_3_), 5.61 (s, 2H, CCH_2_), 5.61 (s, 2H, NNH_2_), 8.16 (s, 1H, N = CH), 13.64 (s, S-*H*). 100 MHz (^13^C-NMR, DMSO-*d*_6_, δ/ppm): 27 (CH_3_), 29.58 (CH_3_), 40.60, (NCH_2_), 106.24, 143.46, 148.22, 148.36, 167.08, (Ar-C), 150.97, 154.49 (2C = O).

#### General procedure for the synthesis of azomethine derivatives of acefylline tethered 4-amino-1,2,4-triazole (7a-k)

A solution of acefylline-based amino triazole (0.1 g, 0.00032 mol) and respective aromatic aldehyde (0.06 g, 0.00064 mol) were refluxed for 6h in ethanol with 1–2 drops of acetic acid. After reaction completion the reaction mixture was cooled overnight. The solid product was afforded by the filtration of reaction mixture followed by recrystallization.

#### (*E*)-7-((4-(benzylideneamino)-5-mercapto-4*H*-1,2,4-triazol-3-yl)methyl)-1,3-dimethyl-1*H*-purine-2,6(3*H*,7*H*)-dione (7a)

Light yellow solid; Yield: 79%; m.p 240°C; IR (KBr): υ 3354 (N-H), 1648 (CO-xanthine), 1545 (C = N), 1476 (Ph), 1455 (C = C), 1334 (C–N). 400 MHz (^1^H-NMR, DMSO-*d*_6_, δ/ppm): 3.16, 3.41 (s, 6H, NCH_3_), 5.80 (s, 2H, NCH_2_), 8.18–8.36 (m, 5H, Ar-H), 8.36, 10.48 (s, 2H, N = CH), 13.66 (s, 1H, SH). 100 MHz (^13^C-NMR, DMSO-*d*_6_, δ/ppm): 27.59 (CH_3_), 29.58 (CH_3_), 40.73, (NCH_2_), 106.30, 124.28, 126.67, 127.12, 128, 129, 132.42, 133.20, 148.56, 149.64 (Ar-C), 147.39, 162.17 (N = C), 151.02, 154.39 (2C = O). MS *m*/*z* (ES^+^) 396.1117 (M^+^) (100%). Anal. Calcd. For C_17_H_16_N_8_O_2_S: C, 51.51; H, 4.07; N, 28.27; Found; C, 52.06; H, 3.27; N, 28.66.

#### (*E*)-7-((4-((2,4-dichlorobenzylidene)amino)-5-mercapto-4*H*-1,2,4-triazol-3-yl)methyl)-1,3-dimethyl-1*H*-purine-2,6(3*H*,7*H*)-dione (7b)

Light yellow solid; Yield: 66%; m.p 235°C; IR (KBr): υ 3354 (N-H), 1648 (CO-xanthine), 1545 (C = N), 1476 (Ph), 1455 (C = C), 1334 (C–N). 400 MHz (^1^H-NMR, DMSO-*d*_6_, δ/ppm): 3.16, 3.41 (s, 6H, NCH_3_), 5.80 (s, 2H, NCH_2_), 8.18 (d, *J*_5_^’^_,6_^’^ = 6 Hz, 1H, H-5^’^), 8.36 (s, 1H, H-3^’^), 8.18 (d, *J*_6_^’^_,5_^’^ = 6.4 Hz, 1H, H-6^’^), 8.36, 10.48 (s, 2H, N = CH), 13.95 (s, 1H, SH). 100 MHz, (^13^C-NMR, DMSO-*d*_6_, δ/ppm): 27.59 (CH_3_), 29.58 (CH_3_), 40.73, (NCH_2_), 106.30, 124.28, 126.67, 127.12, 128, 129, 132.42, 133.20, 148.56, 149.64 (Ar-C), 147.39, 162.17 (N = C), 151.02, 154.39 (2C = O). MS *m*/*z* (ES^+^) 464.0337 (M^+^) (100%). Anal. Calcd. For C_17_H_14_Cl_2_N_8_O_2_S: C, 43.88; H, 3.03; N, 24.08; Found; C, 42.26; H, 3.17; N, 23.66.

#### (*E*)-7-((4-((4-(dimethylamino)benzylidene)amino)-5-mercapto-4*H*-1,2,4-triazol-3-yl)methyl)-1,3-dimethyl-1*H*-purine-2,6(3*H*,7*H*)-dione (7c)

Pink solid; Yield: 72%; m.p 215°C; IR (KBr): υ 3354 (N-H), 1648 (CO-xanthine), 1545 (C = N), 1476 (Ph), 1455 (C = C), 1334 (C–N). 400 MHz (^1^H-NMR, DMSO-*d*_6_, δ/ppm): 1.23 (s, 6H, NCH_3_), 3.14, 3.39 (s, 6H, NCH_3_), 5.76 (s, 2H, NCH_2_), 7.38 (d, *J*_3_^’^_,2_^’^ = *J*_5_^’^_,6_^’^ 6Hz, 2H, H-3^’^and H-5^’^), 7.78 (d, *J*_2_^’^_,3_^’^ = *J*_6_^’^_,5_^’^ 8Hz, 2H, H-2^’^and H-6^’^), 8.66, 9.91 (s, 2H, N = CH), 14.02 (s, 1H, SH). 100 MHz (^13^C-NMR, DMSO-*d*_6_, δ/ppm): 23.70 (CH_3_), 27.54 (CH_3_), 41.36, (NCH_2_), 106.36, 111.59, 111.19, 123.34, 126.67, 125.98, 143.30, 146.91, 148.56, 161.36 (Ar-C), 146.91, 163.67 (N = C), 152.13, 154.8 (2C = O). MS *m*/*z* (ES^+^) 439.1539 (M^+^) (100%). Anal. Calcd. For C_19_H_21_N_9_O_2_S: C, 51.92; H, 4.82; N, 28.68; Found; C, 51.26; H, 4.87; N, 28.46.

#### (*E*)-7-((4-(((2-hydroxynaphthalen-1-yl)methylene)amino)-5-mercapto-4*H*-1,2,4-triazol-3-yl)methyl)-1,3-dimethyl-1*H*-purine-2,6(3*H*,7*H*)-dione (7d)

Light brown solid; Yield: 69%; m.p 120°C; IR (KBr): υ 3354 (N-H), 1648 (CO-xanthine), 1545 (C = N), 1476 (Ph), 1455 (C = C), 1334 (C–N). 400 MHz (^1^H-NMR, DMSO-*d*_6_, δ/ppm): 3.15, 3.39 (s, 6H, NCH_3_), 5.68 (s, 2H, NCH_2_), 5.77 (s, 1H, OH), 7.24 (d, *J*_3_^’^_,4_^’^ = 8Hz, 1H, H-3^’^), 7.41–7.60 (m, 2H, H-6 and H-7), 8.12 (d, *J*_5, 6_ = *J*_7, 8_ 4Hz, 2H, H-5 and H-8), 8.90 (d, *J*_4_^’^_,3_^’^ = 4Hz, 1H, H-4^’^), 7.86, 10.81 (s, 2H, N = CH), 12.0 (s, 1H, SH). 100 MHz (^13^C-NMR, DMSO-*d*_6_, δ/ppm): 27.54 (CH_3_), 29.51 (CH_3_), 40.55, (NCH_2_), 106.19, 112.50, 118.83, 122.24, 124.30, 127.64, 128.90, 129.36, 131.79, 138.48, 143.38, 164.06, 167.06, 192.94 (14 Ar-C), 148.14, 148.35 (N = C), 150.97, 154.29 (2C = O). MS *m*/*z* (ES^+^) 462.1223 (M^+^) (100%). Anal. Calcd. For C_21_H_18_N_8_O_3_S: C, 55.54; H, 3.92; N, 24.23; Found; C, 55.26; H, 3.287; N, 23.96.

#### (*E*)-7-((4-((4-bromobenzylidene)amino)-5-mercapto-4*H*-1,2,4-triazol-3-yl)methyl)-1,3-dimethyl-1*H*-purine-2,6(3*H*,7*H*)-dione (7e)

Cream solid; Yield: 78%; m.p 256°C; IR (KBr): υ 3354 (N-H), 1648 (CO-xanthine), 1545 (C = N), 1476 (Ph), 1455 (C = C), 1334 (C–N). 400 MHz (^1^H-NMR, DMSO-*d*_6_, δ/ppm): 3.16, 3.41 (s, 6H, NCH_3_), 5.80 (s, 2H, NCH_2_), 8.18 (d, *J*_3_^’^_,2_^’^ = *J*_5_^’^_,6_^’^ 6Hz, 2H, H-3^’^and H-5^’^), 8.36 (d, *J*_2_^’^_,3_^’^ = *J*_6_^’^_,5_^’^ 8Hz, 2H, H-2^’^and H-6^’^), 8.36, 10.48 (s, 2H, N = CH), 13.95 (s, 1H, SH). 100 MHz (^13^C-NMR, DMSO-*d*_6_, δ/ppm): 27.59 (CH_3_), 29.58 (CH_3_), 40.73, (NCH_2_), 106.30, 124.28, 126.67, 127.12, 128, 129, 132.42, 133.20, 148.56, 149.64 (Ar-C), 147.39, 162.17 (N = C), 151.02, 154.39 (2C = O). MS *m*/*z* (ES^+^) 474.0222 (M^+^) (100%). Anal. Calcd. For C_17_H_15_BrN_8_O_2_S: C, 42.96; H, 3.18; N, 23.57; Found; C, 42.26; H, 3.27; N, 23.66.

#### (*E*)-7-((5-mercapto-4-((4-methoxybenzylidene)amino)-4H-1,2,4-triazol-3-yl)methyl)-1,3-dimethyl-1*H*-purine-2,6(3*H*,7*H*)-dione (7f)

Light pink solid; Yield: 67%; m.p 168°C; IR (KBr): υ 3354 (N-H), 1648 (CO-xanthine), 1545 (C = N), 1476 (Ph), 1455 (C = C), 1334 (C–N). 400 MHz (^1^H-NMR, DMSO-*d*_6_, δ/ppm): 3.33 (s, 6H, NCH_3_), 3.86 (s, 3H, Ar-OCH_3_), 5.75 (s, 2H, NCH_2_), 7.08 (d, *J*_3_^’^_,2_^’^ = *J*_5_^’^_,6_^’^ 6Hz, 2H, H-3^’^and H-5^’^), 7.81 (d, *J*_2_^’^_,3_^’^ = *J*_6_^’^_,5_^’^ 8Hz, 2H, H-2^’^and H-6^’^), 8.15, 9.78 (s, 2H, N = CH), 13.96 (s, 1H, SH). 100 MHz (^13^C-NMR, DMSO-*d*_6_, δ/ppm): 27.93 (CH_3_), 29.90 (CH_3_), 41.36, (NCH_2_), 56.18 (Ar-OCH_3_), 106.35, 114.59, 120.19, 123.34, 126.67, 125.98, 126.67, 127.12, 132.42, 139.20, 148.56, 149.76, 146.41, 161.67 (14 Ar-C), 146.52, 149.66 (N = C), 151.20, 154.83 (2C = O). MS *m*/*z* (ES^+^) 426.1223 (M^+^) (100%). Anal. Calcd. For C_18_H_18_N_8_O_3_S: C, 50.70; H, 4.25; N, 26.28; Found; C, 50.26; H, 4.27; N, 26.66.

#### (*E*)-7-((4-((4-isopropylbenzylidene)amino)-5-mercapto-4*H*-1,2,4-triazol-3-yl)methyl)-1,3-dimethyl-1*H*-purine-2,6(3*H*,7*H*)-dione (7g)

Light brown solid; Yield: 73%; m.p 160°C; IR (KBr): υ 3354 (N-H), 1648 (CO-xanthine), 1545 (C = N), 1476 (Ph), 1455 (C = C), 1334 (C–N). 400 MHz (^1^H-NMR, DMSO-*d*_6_, δ/ppm): 1.20 (s, 6H, CCH_3_), 2.92 (s,1H, CHCH_3_), 3.14, 3.39 (s, 6H, NCH_3_), 5.76 (s, 2H, NCH_2_), 7.38 (d, *J*_3_^’^_,2_^’^ = *J*_5_^’^_,6_^’^ 6Hz, 2H, H-3^’^and H-5^’^), 7.78 (d, *J*_2_^’^_,3_^’^ = *J*_6_^’^_,5_^’^ 8Hz, 2H, H-2^’^and H-6^’^), 8.16, 9.91 (s, 2H, N = CH), 14 (s, 1H, SH). 100 MHz (^13^C-NMR, DMSO-*d*_6_, δ/ppm): 23.3 (2CH_3_), 27.93 (CH_3_), 29.90 (CH_3_), 33.2 (CH), 41.36, (NCH_2_), 106.36, 126.58, 126.97, 127.70, 128.50, 128.91, 148.12, 150.96, 152.13, 161.35 (Ar-C), 146.52, 163.67 (N = C), 151.20, 153.99 (2C = O). MS *m*/*z* (ES^+^) 438.1586 (M^+^) (100%). Anal. Calcd. For C_20_H_22_N_8_O_2_S: C, 54.78; H, 5.06; N, 25.55; Found; C, 54.26; H, 5.27; N, 25.66.

#### (*E*)-7-((5-mercapto-4-((4-nitrobenzylidene)amino)-4*H*-1,2,4-triazol-3-yl)methyl)-1,3-dimethyl-1H-purine-2,6(3*H*,7*H*)-dione (7h)

Yellow solid; Yield: 69%; m.p 242°C; IR (KBr): υ 3354 (N-H), 1648 (CO-xanthine), 1545 (C = N), 1476 (Ph), 1455 (C = C), 1334 (C–N). 400 MHz (^1^H-NMR, DMSO-*d*_6_, δ/ppm): 3.16, 3.41 (s, 6H, NCH_3_), 5.80 (s, 2H, NCH_2_), 8.18 (d, *J*_3_^’^_,2_^’^ = *J*_5_^’^_,6_^’^ 6Hz, 2H, H-2^’^and H-6^’^), 8.36 (d, *J*_2_^’^_,3_^’^ = *J*_6_^’^_,5_^’^ 8Hz, 2H, H-3^’^and H-5^’^), 10.48 (s, 2H, N = CH), 13.66 (s, 1H, SH). 100 MHz (^13^C-NMR, DMSO-*d*_6_, δ/ppm): 27.53 (CH_3_), 29.58 (CH_3_), 40.68, (NCH_2_), 106.30, 124.33, 129, 138.11, 143.44, 148.09, 148.36, 148.62, 149.64, 162.67 (Ar-C), 147.42, 149.32 (N = C), 151.20, 154.83 (2C = O). MS *m*/*z* (ES^+^) 441.0968 (M^+^) (100%). Anal. Calcd. For C_17_H_15_N_9_O_4_S: C, 46.26; H, 3.43; N, 28.56; Found; C, 46.22; H, 3.27; N, 28.66.

#### (*E*)-7-((5-mercapto-4-((2-nitrobenzylidene)amino)-4*H*-1,2,4-triazol-3-yl)methyl)-1,3-dimethyl-1*H*-purine-2,6(3*H*,7*H*)-dione (7i)

White solid; Yield: 71%; mp 240°C; IR (KBr): υ 3354 (N-H), 1648 (CO-xanthine), 1545 (C = N), 1476 (Ph), 1455 (C = C), 1334 (C–N). 400 MHz (^1^H-NMR, DMSO-*d*_6_, δ/ppm): 3.32, 3.39 (s, 6H, NCH_3_), 5.80 (s, 2H, NCH_2_), 7.18–8.36 (m, 4H, Ar-H), 10.48 (s, 2H, N = CH), 13.66 (s, 1H, SH). 100 MHz (^13^C-NMR, DMSO-*d*_6_, δ/ppm): 27.53 (CH_3_), 29.58 (CH_3_), 40.68, (NCH_2_), 106.30, 124.33, 129, 138.11, 143.44, 148.09, 148.36, 148.62, 149.64, 162.67 (Ar-C), 147.42, 149.32 (N = C), 151.20, 154.83 (2C = O). MS *m*/*z* (ES^+^) 441.0968 (M^+^) (100%). Anal. Calcd. For C_17_H_15_N_9_O_4_S: C, 46.26; H, 3.43; N, 28.56; Found; C, 46.15; H, 3.27; N, 28.66.

#### Synthesis of (*E*)-7-((4-((2-hydroxybenzylidene)amino)-5-mercapto-4*H*-1,2,4-triazol-3-yl)methyl)-1,3-dimethyl-1*H*-purine-2,6(3*H*,7*H*)-dione (7j)

Off white solid; Yield: 62%; m.p 232°C; IR (KBr): υ 3354 (N-H), 1648 (CO-xanthine), 1545 (C = N), 1476 (Ph), 1455 (C = C), 1334 (C–N). 400 MHz (^1^H-NMR, DMSO-*d*_6_, δ/ppm): 3.15, 3.40 (s, 6H, NCH_3_), 5.35 (s, 1H, OH), 5.75 (s, 2H, NCH_2_), 6.97–7.98 (m, 4H, Ar-H), 8.15, 10.21 (s, 2H, N = CH), 13.96 (s, 1H, SH). 100 MHz (^13^C-NMR, DMSO-*d*_6_, δ/ppm): 27.57 (CH_3_), 29.54 (CH_3_), 40.77, (NCH_2_), 106.34, 116.71, 118.28, 119.57, 126.90, 134.53, 143.39, 148.15, 159.91, 161.67 (Ar-C), 147.03, 162.03 (N = C), 150.99, 154.39 (2C = O). MS *m*/*z* (ES^+^) 412.1066 (M^+^) (100%). Anal. Calcd. For C_17_H_16_N_8_O_3_S: C, 49.51; H, 3.91; N, 27.17; Found; C, 49.26; H, 3.87; N, 27.66.

#### (*E*)-7-((4-((4-bromo-2-hydroxybenzylidene)amino)-5-mercapto-4*H*-1,2,4-triazol-3-yl)methyl)-1,3-dimethyl-1*H*-purine-2,6(3*H*,7*H*)-dione (7k)

Off white solid; Yield: 76%; m.p 213°C; IR (KBr): υ 3354 (N-H), 1648 (CO-xanthine), 1545 (C = N), 1476 (Ph), 1455 (C = C), 1334 (C–N). 400 MHz (^1^H-NMR, DMSO-*d*_6_, δ/ppm): 3.15, 3.40 (s, 6H, NCH_3_), 5.35 (s, 2H, NCH_2_), 5.75 (s, 1H, OH), 6.97–7.98 (m, 3H, Ar-H), 8.15, 10.21 (s, 2H, N = CH), 13.96 (s, 1H, SH). 100 MHz (^13^C-NMR, DMSO-*d*_6_, δ/ppm): 27.57 (CH_3_), 29.54 (CH_3_), 40.77, (NCH_2_), 106.34, 116.71, 118.28, 119.57, 126.90, 134.53, 143.39, 148.15, 159.91, 161.67 (Ar-C), 147.03, 162.03 (N = C), 150.99, 154.39 (2C = O). MS *m*/*z* (ES^+^) 490.0171 (M^+^) (100%). Anal. Calcd. For C_17_H_15_BrN_8_O_3_S: C, 41.56; H, 3.08; N, 22.81; Found; C, 41.26; H, 3.17; N, 22.66.

### MTT cell growth assay

#### Cell Culture and treatment

Hep G2 cells from hepatocellular carcinoma were cultured in 100 mL of DMEM medium supplemented with penicillin (100 μg/mL), streptomycin (100 μg/mL), fetal bovine serum (FBS) (10%) and incubated them in humid air containing carbon dioxide (5%), at 37°C. Solutions of synthesized derivatives were made using dimethyl sulfoxide (DMSO) as solvent. The sample solutions were diluted with DMSO up to a final concentration of 0.05%. In all experiments DMSO was used to treat the cells of control group.

#### Cell Viability determination using MTT assay

By using cell growth inhibition MTT assay as standard [41], cell viability was evaluated. The cancer cells were seeded and plated overnight to grow at 96-well trays. Further the cultured cells were incubated for 4 h at 37° C after treatment of cells with culture medium and synthesized series of compounds at different concentrations for 48 h. After that the cell growth was accessed by addition of 500 μg mL^-1^ MTT reagent to each well followed by incubation for 4h. After incubation the medium was removed and 150 μL of DMSO was added to each well. The absorbance of the each well was observed at 490 nm using plate reader for the evaluation of percentage cell viability.

### Hemolysis assay

Hemolytic activity of the synthesized novel candidates have been evaluated through the method reported in literature [42]. The samples of blood (3 mL) were taken of albino rats (any genus) in heparin containing tubes and centrifuged at 1000 × g for 5 min. The supernatant was poured off and isolated RBC’s were washed three times with 5 mL of chilled sterile phosphate buffer saline (PBS) having pH = 7.4. A diluted RBC’s suspension of concentration 180 μL was made in chilled PBS. The synthesized series of compounds solution of concentration 20 μL in DMSO were added to 180 μL of diluted RBC’s suspension which were further incubated for 30 min at 37°C. Further, tubes were placed in ice (5 min) followed by centrifugation (5 min). After centrifugation supernatant was removed and diluted the solution up to 10 times with cold PBS. The DMSO was the negative control while ABTS was used as positive control, respectively.

Percentage hemolysis was determined using following formula by determining the absorption of samples (at 576 nm).


$$\%\;age\;hemolysis=\frac{Absorbance\;of\;sample–Absorbance\;of\;negative\;control\;(DMSO)}{Absorbance\;of\;positive\;control\;(ABTS)}\times 100$$

### Thrombolysis assay

The synthesized novel candidate’s thrombolytic activity was evaluated by using proposed method [43]. The samples of blood (1 mL) from rats (any sex) were transferred to pre-weighed eppendorfs followed by incubation for 45 min at 37°C. After the clot formation the serum was removed from eppendorfs and weight of clot was determined by subtracting the weight of empty eppendrof from the eight of eppendrof containing clot. In the clot containing eppendorphs, sample solutions of synthesized compounds with concentration of 100 μL were added followed by incubation for 3 h at 37°C. After incubation DMSO as negative control was added to the eppendorphs. Excessive fluid was removed from the sample tubes after clot lysis and weight of the eppendorphs was determined. The assay used ABTS as positive control. From the weight after clot lysis percentage of clot lysis was determined using following expression:

$$Percentage\;of\;clot\;lysis\;(\%)=\frac{Initial\;weight\;of\;clot\;‐Final\;weight\;of\;clot}{Initial\;clot\;weight}\times 100$$

### Molecular docking method

Molecular docking study was performed to access a comparative analysis of the activities of the series of candidates (**7a-k**). The docking calculations provide evidence about the molecule’s interactions and activities. Each parameter explains different chemical and biological property of the molecule [44,45].

Docking calculations of the title molecules was carried on Maestro Molecular modeling platform (version 12.2) by Schrödinger [46]. Preparation of title compounds and target proteins is required before molecular docking analysis and calculations. This molecular modeling platform combines many modules for final assessment using the Gaussian software program [47]. First of all optimized structures of the compounds as well as target proteins are required to observe the binding modes, interactions and evaluation of activities by performing calculations. The module used for the optimized structures was “LigPrep” [48]. Afterwards, the protein preparation module [49] was used for the preparation of proteins for calculations. After obtaining the optimized structures of title compounds and proteins, they were allowed to process *via* docking analysis to interact using Glide ligand module [50]. This module uses OPLS3e method for the docking analysis and performing calculations. Further, ADME/T analysis was carried out using “Qik-prop module” [51]. to evaluate the possibility of the synthesized agents to be potential drugs for future applications in pharmaceutical industry.

## Results and discussion

### Chemistry

**Fig 2:**
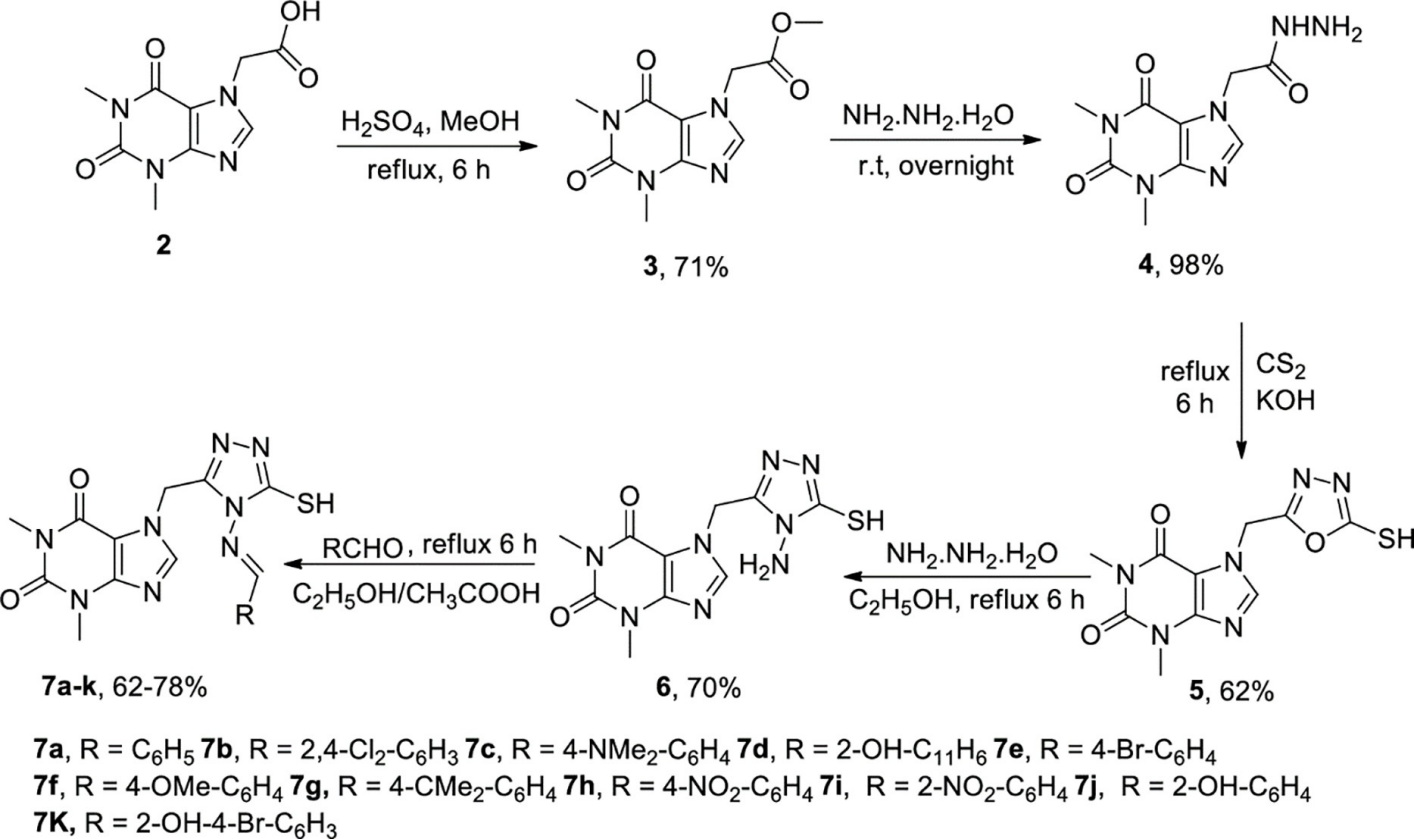
Pathway and reaction conditions for the synthesis of compounds (7a-k).

The synthesis of ester **3** *via* Fisher esterification of **2 (**acefylline) with methanol and catalytic amount of H_2_SO_4_ under reflux conditions was performed and was obtained smoothly in 71% yield. In the next step, methyl ester of theophylline **3** was transformed to theophylline-7-acetohydrazide **4** (98% yield) by reacting with hydrazine monohydrate in the presence of methanol as solvent. Theophylline-7-acetohydrazide **4** was further treated with CS_2_ using KOH under 6 h reflux to synthesize theophylline oxadiazole hybrid **5** in 62% yield [30]. Further, amino substituted triazole **6** was afforded in 70% yield by treatment of the theophylline oxadiazole hybrid **5** with hydrazine monohydrate in ethanol solvent at reflux for 6 h. Finally, various 4-amino-1,2,4-triazole-acefylline substituted azomethine analogs **7a-k** (Fig 2) were synthesized in a good yield (62–78%) by treatment of amino substituted triazole **6** with various aldehydes in the presence of acetic acid under reflux in ethanol for 6 h [52].

### Spectral explanation of demonstrative molecule (7h)

**Fig 3:**
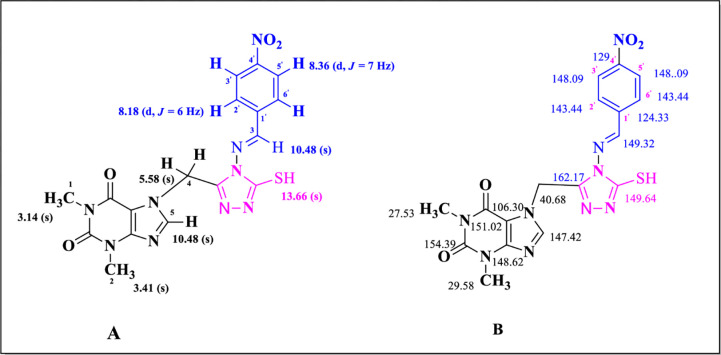
^1^H-NMR and ^13^C-NMR analysis of compound **7h**, (A) and (B), respectively.

Molecule **7h** was synthesized as a yellow solid and its structure was confirmed by MS-EI spectrum (M^+^) at m/z 441.0968 and further confirmed by ^1^H- and ^13^C-NMR and IR. To pronounce several functional groups in FT-IR, absorption peaks of the compound were detected at υ 3354 (N–H., str); 1648 (CO-xanthene., str); 1545 (C = N., str); Ph (1476); 1455 (C = C., str); 1334 (C–N., str); 804 (C–H); 689 (S–C) cm^-1.^ Two downfield signals were detected in the ^1^H-NMR spectrum at δ 10.48 ppm for azomethine and N-H of the xanthene ring. Two protons of CH_2_ vibrated at δ 5.80 ppm in the up-field region whereas six protons of purine ring vibrated as a singlet at δ 3.16 ppm and δ 3.41 ppm. Two protons of aromatic ring (*H*-2’ and *H*-6’) resonated at δ 8.18 ppm (*J* = 6 Hz) while the spectra of *H*-3’ and *H*-5’ were appeared as a doublet at δ 8.36 ppm (*J* = 8 Hz) **(Fig 3A)**.

The carbon framework of all the 17 carbons in **7h** was also verified by ^13^C-NMR. The two signals of 2C = O at δ 151.02 ppm and 154.39 ppm belonged to the purine ring while the other two downfield signals were shown by quaternary carbon at δ 162.17 ppm and 149.64 ppm confirmed the presence of 1,2,4-triazole ring. A signal observed at δ 147.42 ppm and two signals at δ 106.30 ppm and 148.62 ppm confirmed the presence of methine and C = C, respectively, in theophylline ring. A signal of methylene linker between purine and triazole ring was detected at δ 40.68 ppm. The *para* nitrophenyl ring attached with azomethine showed two methine signals at δ 148.09 ppm and δ 148.36 ppm, the other two signals appeared at δ 143.44 ppm and δ 138.11 ppm. While the signals of phenyl carbon for C-NO_2_ appeared at δ 124.33 ppm and for C-C = N appeared at δ 129 ppm. The presence of azomethine functionality was justified by downfield signal of N = C at δ 149.32 ppm **(Fig 3B)**. The other compounds of the synthesized series (**7a-k**) were verified similarly.

### Anti-cancer activity

The cytotoxic potential of all the synthesized derivatives **7a-k** was studied by MTT (3-(4,5-dimethylthiazol-2-yl)-2,5-diphenyl-2H-tetrazolium bromide) assay against cancer cell line Hep G2 (liver). In general the compounds having phenyl rings with electron donating substituents showed greater activity. Compounds **7d** (cell viability = 11.71 ± 0.39%) and **7g** (cell viability = 24.20 ± 1.34%) were more effective against liver cancer cell line (Hep G2). Compound **7j** (cell viability = 32.45 ± 1.35%) also showed greater activity as compared to acefylline (cell viability = 80 ± 3.87%) using concentration (100μg/μl) of compound (Table 1). Compounds **7c** (cell viability = 52.18 ± 5.25%) and **7k** (cell viability = 59.73 ± 3.47%) showed moderate activity. While compounds **7a** (cell viability = 80.19 ± 5.06%), **7b** (cell viability = 92.97 ± 4.47%), **7e** (cell viability = 45.73 ± 0.64%), **7f** (cell viability = 54.48 ± 6.13%), **7h** (cell viability = 62.26 ± 1.18%) and **7i** (cell viability = 67.66 ± 0.25%) were considered the least active against cancer with comparatively high cell viability values.

**Table 1:** Anti-cancer, hemolytic, and thrombolytic activity of target compounds 7a-k.

Compounds	R	*Cell viability Hep G2	% Hemolysis	% Thrombolysis
**7a**	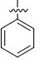	80.19 ± 4.47	8.49	45
**7b**	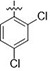	92.97 ± 5.06	5.1	49.9
**7c**	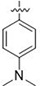	72.18 ± 5.56	4.5	42
**7d**	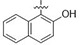	11.71 ± 0.39	34.7	42.7
**7e** **7f**		45.73 ± 0.64 54.48 ± 6.13	17.9 30.9	49 30.9
**7g**	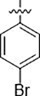	24.20 ± 1.34	0.26	48.2
**7h**	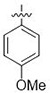	62.26 ± 1.18	6.86	49.1
**7i**	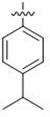	67.66 ± 0.25	1.76	46.3
**7j**	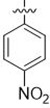	32.45 ± 1.35	2.98	39.4
**7k**	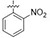	59.73 ± 3.47	19.7	52.1
Acefylline	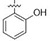	80 ± 3.87	44.08	6.85
DMSO	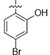	100 ± 0	0.01	0.57
ABTS			95.5	88

*Cell viability: (Mean ± SD (standard deviation), in triplicate with concentration (100 μg/mL).

### Structure-activity relationship (SAR)

**Fig 4:**
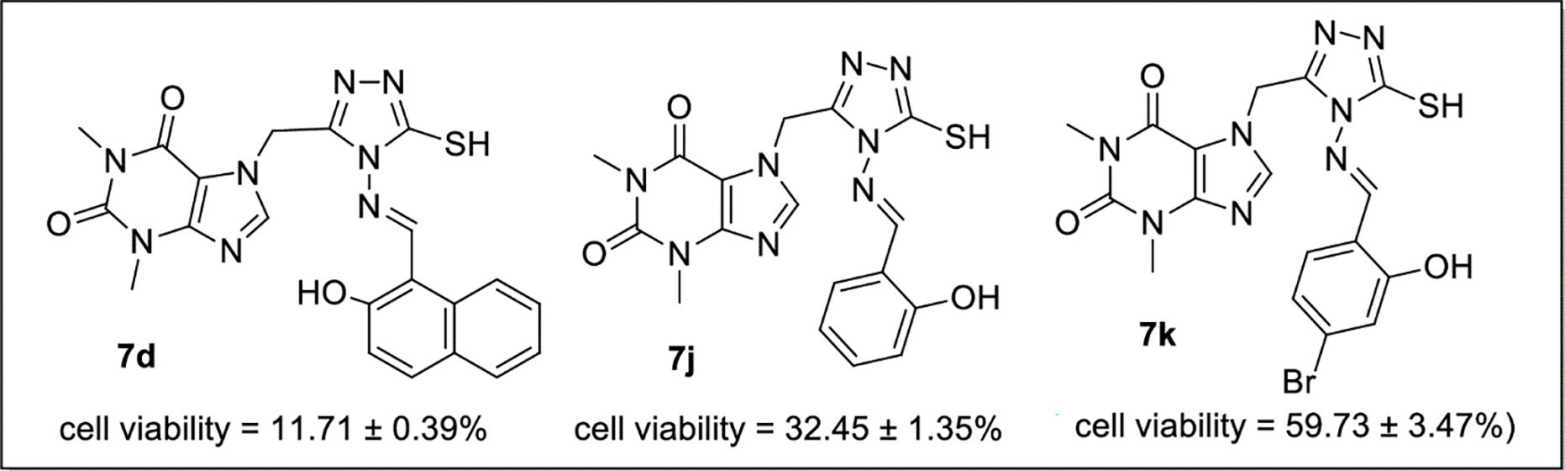
SAR of 7d, 7j and 7k.

Structure-activity relationship of synthesized novel compounds was examined by variation of substituents on phenyl ring scaffold present in azomethine derivatives of acefylline-triazole hybrid to get comprehensive evidence about the anti-proliferative activity of the synthesized derivatives. The compound having unsubstituted phenyl ring exhibited relatively low activity e.g., compound **7a** possessing unsubstituted phenyl ring (cell viability = 80.19 ± 5.06%). The Structure-activity relationship of compounds revealed that incorporation of electron-donating substituents increases anti-cancer activity e.g., the compound **7d** having a hydroxyl group at naphthyl ring was found to induce greater inhibitory potential towards Hep G2 cell line and exhibited pronounced anticancer activity (cell viability = 11.71 ± 0.39%), the activity of compound **7j** (cell viability = 32.45 ± 1.35%) bearing hydroxyl group at phenyl ring was decreased which shows that activity of the compounds increases with aromaticity (Fig 4). However, the activity of derivative **7k** (cell viability = 59.73 ± 3.47%) having both electron-donating as well as electron-withdrawing substituents at phenyl ring is decreased. This shows that *ortho* substituted phenyl ring with electron-donating groups compared to its para analog have preferable orientation for interactions with binding sites and stands for their potent activities.

**Fig 5:**
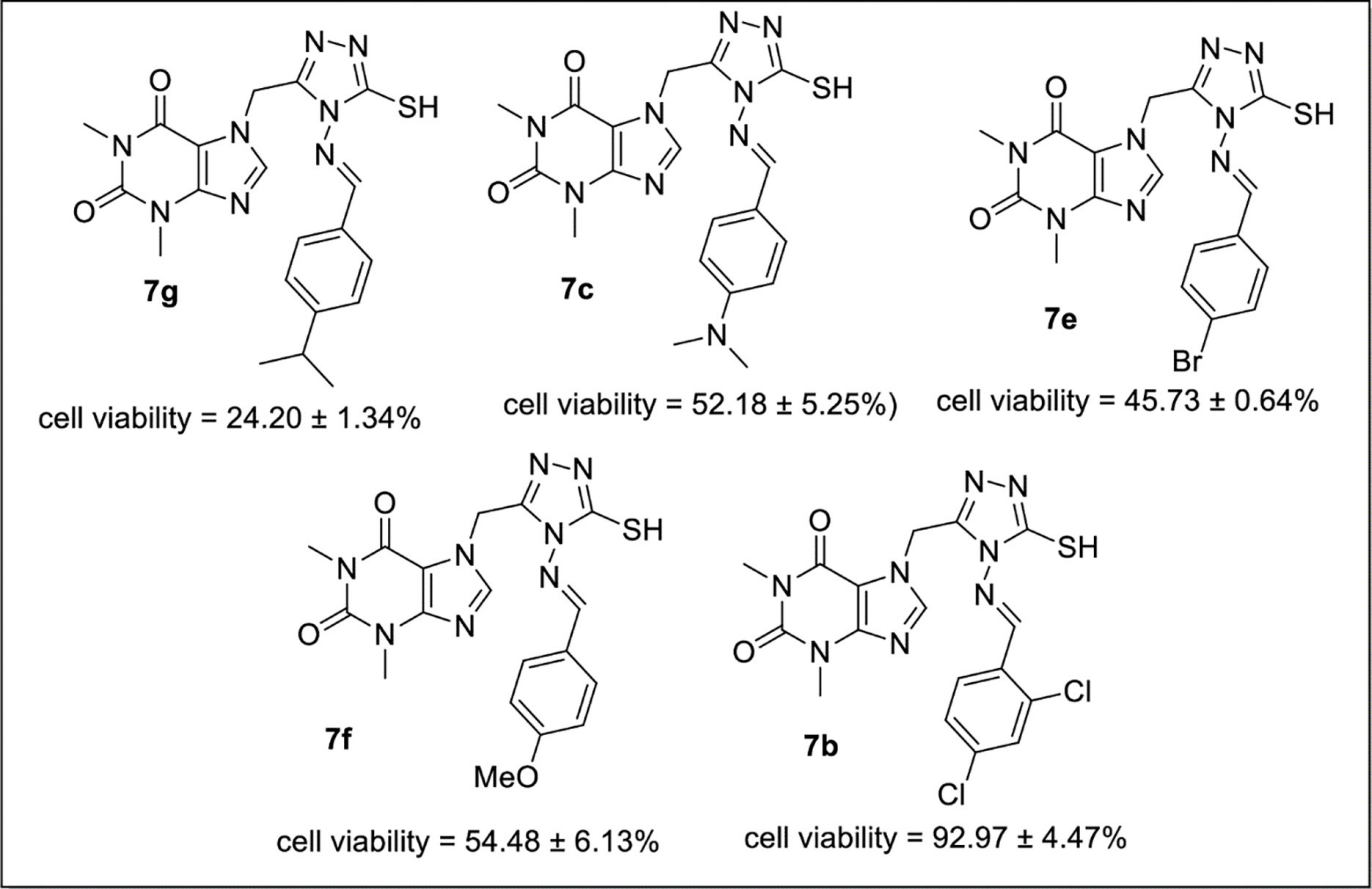
SAR of 7g, 7c, 7e, 7f and 7b.

Compound **7g** having *p*-isopropyl substituted phenyl ring (100μg/μl concentration) exhibited greater activity (cell viability = 24.20 ± 1.34%). Similarly, compound **7c** (cell viability = 52.18 ± 5.25%), **7e** (cell viability = 45.73 ± 0.64%) and **7f** (cell viability = 54.48 ± 6.13%) having *p-*dimethylamino tethered, bromo and methoxy substituent at para position of pheny ring exhibited moderate anticancer activity in comparison to the reference drug acefylline (cell viability = 86.32 ± 11.75%). While, compound **7b** (cell viability = 92.97 ± 4.47%) with two chloro groups on phenyl ring at *para* and *ortho* positions was the least active derivative of the series (Fig 5).

**Fig 6:**
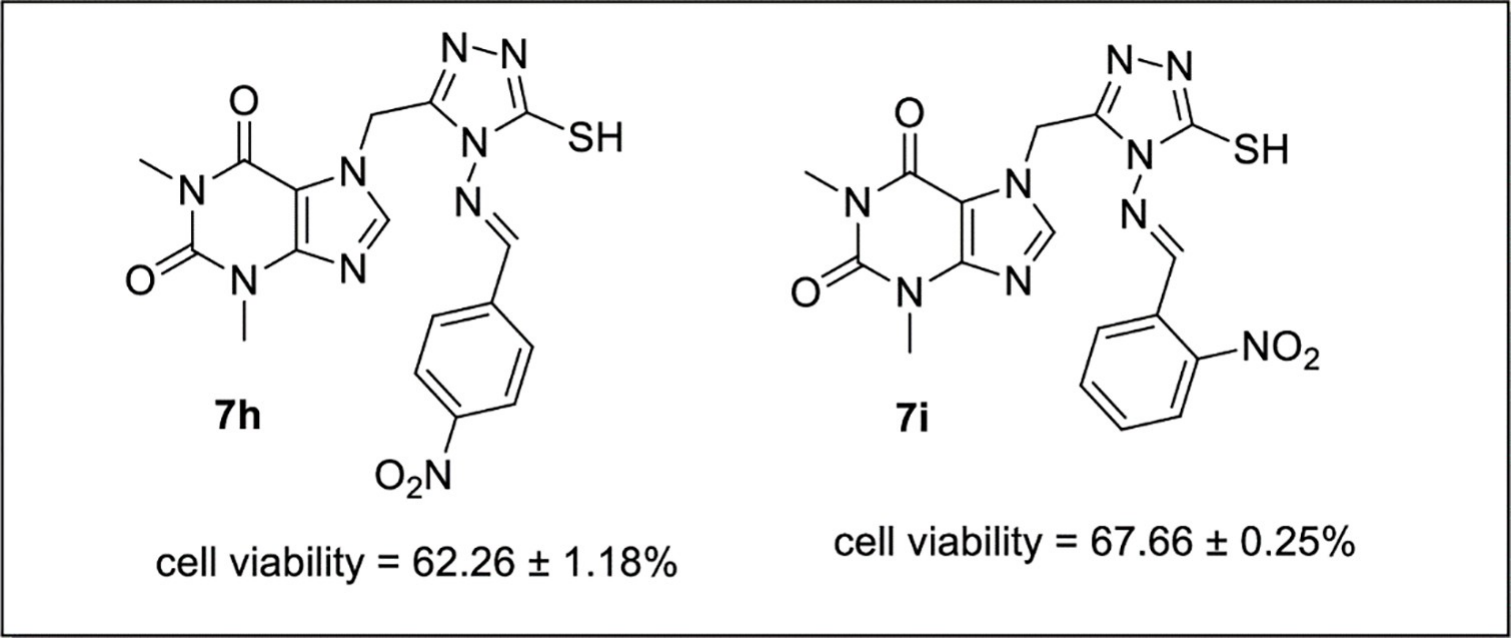
SAR of 7h and 7i.

Compounds **7h** (cell viability = 62.26 ± 1.18%) having nitro group on phenyl ring at para position displayed moderate activity whereas activity of derivative **7i** (cell viability = 67.66 ± 0.25%) was slightly decreased bearing nitro substituent at *ortho* position (Fig 6).

### Hemolytic activity

The Hemolytic activity of novel candidates (**7a-k)** was tested and they were found to induce poor to moderate hemolysis (**Table 1** in terms of % age hemolysis). Among these derivatives, the compounds **7g** (0.26%), **7i** (1.76%) and **7j** (2.98%) were found least toxic while, the compounds **7d** (34.7%), **7f** (30.9%), **7k** (19.7%) and **7f** (30.9%) were found highly toxic. Moderate toxicity was exhibited by compounds **7a** (8.49%), **7h** (6.86%), **7b** (5.10%) and **7c** (4.5%).

### Thrombolytic activity

All the synthesized derivatives (**7a-k)** showed moderate clot lysis activity (Table 1). Among the synthesized derivatives, maximum thrombolytic activity was observed in compound **7k** (52.1%). Compounds **7f** (30.9%), **7j** (39.4%), **7c** (42%), **7d** (42.7%), **7a** (45%) and **7i** (46.3%), **7g** (48.2%), **7e** (49%), **7h** (49.1%) and **7b** (49.9%) were found moderately active thrombolytic agent.

### Computational modelling studies

Molecular docking study has been an important technique widely used for virtual screening of compounds to predict their activitiy of molecules against proteins involved in cancer cells [53]. Several parameters along with the values of docking score parameter were accessed to compare the activities of compounds. The molecule with the lowest numerical value (higher negative value) is the most potent compound. The orientation and interaction of the title compound and target protein are determinant of the numerical values of the docking parameters. The polar interactions that account for the activity of the molecules are hydrophobic interactions, π-π interactions, hydrogen bonding, and halogen interactions [54–56].

A number of other parameters were also accessed using the molecular docking studies along with the docking score parameter and each parameter plays a significant contribution to explain various properties of the molecule. These parameters include Glide ligand efficiency, Glide evdw, Glide ecoul, and Glide hbond that explain the molecular properties [54]. It explains many chemical interactions such as Coulomb interactions, H-bonding, and Van der Waals forces that came into action during the interaction between proteins and title molecules. However, parameters such as Glide energy, Glide emodel, Glide einternal, and Glide posenum provide numerical evidence of the orientation of the title compound and proteins interaction [55].

**Fig 7:**
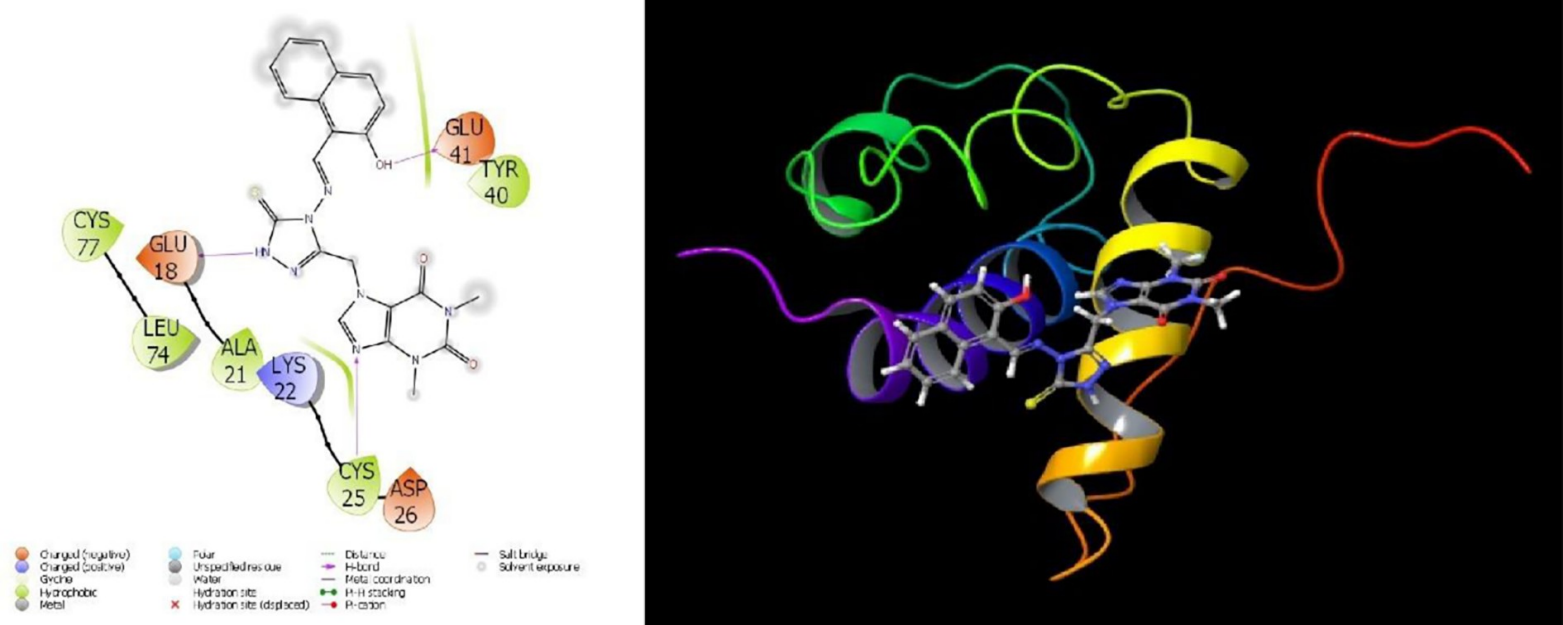
Depiction of title compound 7d interactions with 2H80 protein.

**Fig 8:**
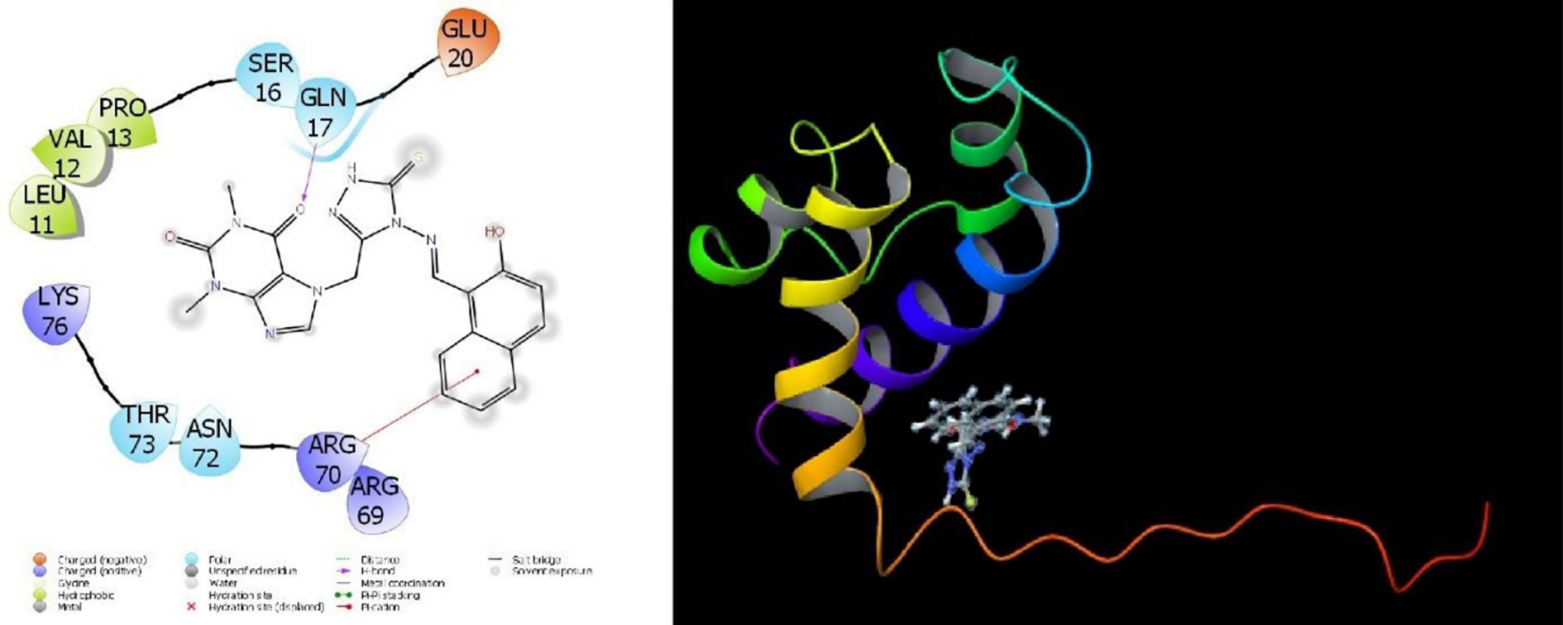
Depiction of title compound 7d interactions with 2JW2 protein.

**Fig 9:**
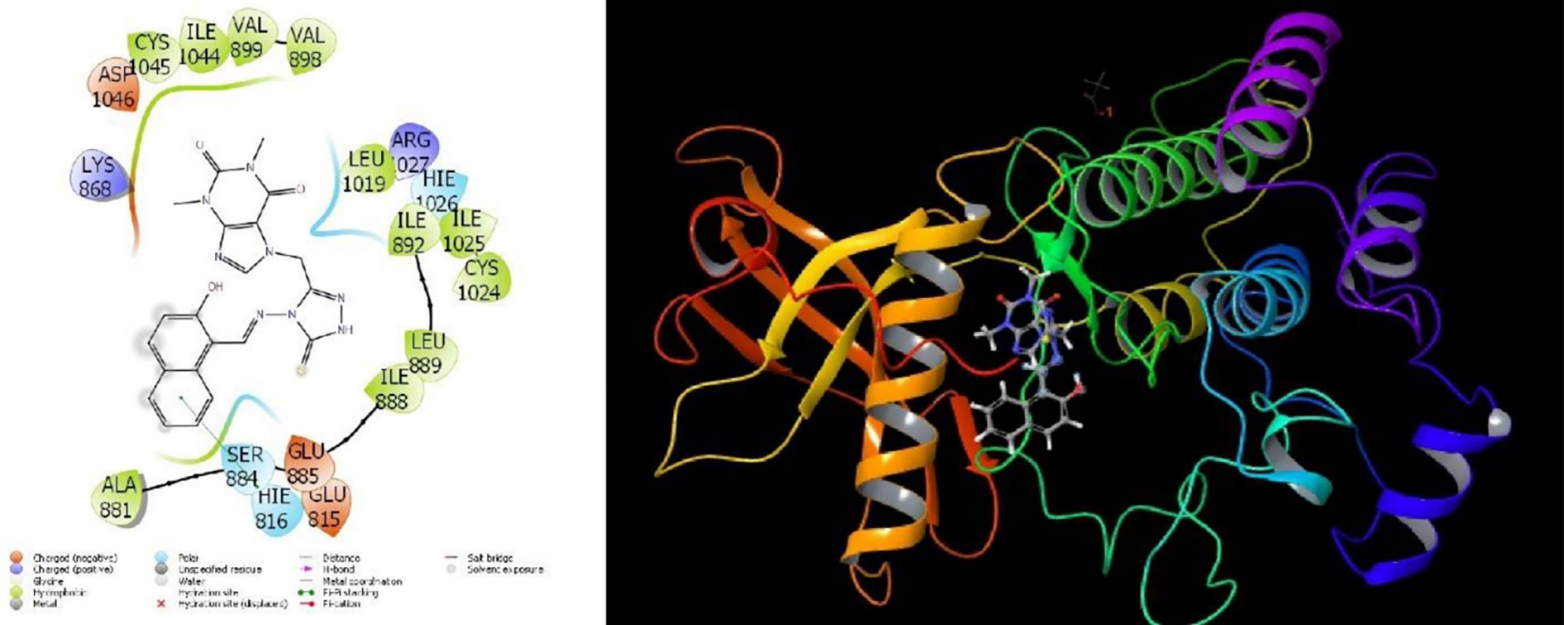
Depiction of compound 7d interactions with 3WZE protein.

Molecular interactions with proteins are given in **Figs 7–9** and the obtained parameters collectively are given in Table 2.

**Table 2:** Docking calculations of title compounds (7a-k) against target proteins.

**3WZE**	**7a**	**7b**	**7c**	**7d**	**7e**	**7f**	**7g**	**7h**	**7i**	**7j**	**7k**	**Acefylline**
Docking score	-5.88	-5.56	-5.53	-6.10	-5.28	-5.18	-5.16	-5.41	-5.56	-5.38	-5.67	-5.85
Glide ligand efficiency	-0.21	-0.19	-0.18	-0.18	-0.18	-0.17	-0.17	-0.17	-0.18	-0.19	-0.19	-0.34
Glide hbond	0.00	0.00	0.00	-0.04	0.00	0.00	0.00	0.00	0.00	0.00	0.00	-0.15
Glide evdw	-48.27	-50.35	-49.88	-51.06	-50.61	-49.22	-43.77	-50.25	-45.23	-42.92	-50.99	-28.95
Glide ecoul	-1.18	-1.65	-2.83	-2.82	0.06	-1.57	-1.79	-1.86	-3.76	-0.69	-1.33	-2.21
Glide emodel	-66.42	-68.47	-69.64	-79.35	-63.58	-63.88	-58.96	-66.89	-63.68	-67.87	-78.42	-43.89
Glide energy	-49.46	-51.99	-52.71	-53.88	-50.55	-50.79	-45.57	-52.11	-48.99	-43.61	-52.32	-31.16
Glide einternal	4.49	3.06	2.07	5.54	6.58	6.52	2.01	5.83	6.31	0.82	2.32	1.58
Glide posenum	96	58	25	119	89	279	114	37	242	103	68	322
**2H80**	**7a**	**7b**	**7c**	**7d**	**7e**	**7f**	**7g**	**7h**	**7i**	**7j**	**7k**	**Acefylline**
Docking score	6.92	-3.79	-2.98	-4.20	-0.17	-2.05	-1.91	-	-3.14	-3.54	-3.16	-
Glide ligand efficiency	0.25	-0.13	-0.10	-0.13	-0.01	-0.07	-0.06	-	-0.10	-0.12	-0.11	-
Glide hbond	-0.61	0.00	-0.24	-0.57	0.00	-0.32	-0.32	-	0.00	-0.56	-0.02	-
Glide evdw	-30.64	-34.63	-28.18	-29.00	-26.59	-25.51	-26.85	-	-28.24	-25.48	-28.87	-
Glide ecoul	-5.35	-2.09	-7.13	-10.00	-3.28	-6.30	-6.19	-	-0.77	-5.30	-4.34	-
Glide emodel	-43.19	-44.20	-41.18	-46.31	-33.02	-37.64	-38.58	-	-32.40	-42.16	-46.39	-
Glide energy	-35.98	-36.72	-35.31	-39.00	-29.87	-31.81	-33.04	-	-29.01	-30.78	-33.21	-
Glide einternal	3.19	1.89	5.95	6.67	3.95	7.28	7.62	-	4.98	2.19	3.59	-
Glide posenum	35	60	16	22	13	13	11	-	31	29	25	-
**2JW2**	**7a**	**7b**	**7c**	**7d**	**7e**	**7f**	**7g**	**7h**	**7i**	**7j**	**7k**	**Acefylline**
Docking score	-3.34	-3.27	-3.74	-3.68	-3.57	-3.99	-4.14	-3.17	-3.53	-3.46	-3.21	-3.23
Glide ligand efficiency	-0.12	-0.11	-0.12	-0.11	-0.12	-0.13	-0.13	-0.10	-0.11	-0.12	-0.11	-0.19
Glide hbond	-0.32	-0.13	-0.32	-0.37	-0.35	-0.32	-0.32	-0.01	-0.61	-0.29	-0.16	-0.30
Glide evdw	-25.70	-29.67	-35.40	-32.23	-26.16	-27.08	-35.40	-31.71	-29.29	-28.35	-28.46	-8.45
Glide ecoul	-5.71	-6.73	-6.87	-7.52	-5.78	-7.08	-7.09	-5.54	-5.98	-4.82	-5.72	-5.85
Glide emodel	-37.75	-41.88	-51.98	-53.83	-38.93	-42.11	-52.25	-43.25	-42.01	-44.79	-46.38	-19.51
Glide energy	-31.41	-36.41	-42.26	-39.76	-31.94	-34.16	-42.48	-37.24	-35.27	-33.17	-34.19	-14.30
Glide einternal	1.67	3.57	2.15	2.30	1.41	2.64	2.30	2.22	2.10	3.13	3.43	0.96
Glide posenum	88	287	197	359	18	80	141	121	344	318	109	71

The docking calculations are just a theoretical method for comparing the activities of compounds. It does not provide any information about the effect, reaction, and toxicity of these molecules if they are taken as drugs into human metabolism, therefore ADME/T analysis is calculated. Many parameters are calculated and given in Table 3, for this calculation each parameter describes a different property of molecules. The first parameters obtained by ADME/T calculations are the chemical properties of molecules such as dipole moment, molar mass, volume, hydrogen bond acceptance, and hydrogen bond donation [56]. The next parameters are biological parameters, which are calculated to predict the orientation and interactions of molecules in human metabolisms, such as QPlogHERG, QPPCaco, QPPMDCK, Human Oral Absorp., Jm [53]. Parameters such as QPPCaco and QPlogBB refer to the brain-blood and gut-blood barriers of molecules, respectively. Other important parameters are RuleOfFive that is Lipinski’s rule of five and RuleOfThree [57]. that is Jorgensen’s rule of three [58–60]. Although the RuleOfFive parameter is known as Lipinski’s rule of five, it consists of four rules, which are mol_MW < 500, QPlogPo/w < 5, donorHB ≤ 5, accptHB ≤ 10. In addition to above, RuleOfThree parameter consists of QPlogS > -5.7, QP PCaco > 22 nm/s, # Primary Metabolites < 7 rules.

**Table 3:** ADME properties of the molecules (7a-k).

	7a	7b	7c	7d	7e	7f	7g	7h	7i	7j	7k	Reference Range
mol_MW	396	465	439	462	475	426	439	441	441	412	491	130–725
dipole (D)	10.9	8.7	11.2	13.2	9.4	11.0	11.4	4.1	8.6	9.2	7.7	1.0–12.5
SASA	670	710	747	732	699	699	748	713	702	678	707	300–1000
FOSA	194	188	343	185	194	283	355	194	188	190	190	0–750
FISA	167	167	173	197	167	167	167	271	255	210	210	7–330
PISA	252	173	173	294	203	192	169	191	207	223	174	0–450
WPSA	56	182	56	55	134	56	56	56	52	54	132	0–175
volume (A^3^)	1182	1263	1331	1321	1235	1250	1337	1262	1252	1201	1255	500–2000
donorHB	0.8	0.8	0.8	1.8	0.8	0.8	0.8	0.8	0.8	1.8	1.8	0–6
accptHB	8.5	8.5	9.5	9.25	8.5	9.25	8.5	9.5	9.5	9.25	9.25	2.0–20.0
glob (Sphere = 1)	0.8	0.8	0.8	0.8	0.8	0.8	0.8	0.8	0.8	0.8	0.8	0.75–0.95
QPpolrz (A^3^)	39.7	42.2	44.3	45.0	41.4	41.2	44.5	41.7	41.5	39.5	41.2	13.0–70.0
QPlogPC16	12.7	13.9	13.7	14.8	13.5	13.0	13.7	13.9	13.7	13.2	14.0	4.0–18.0
QPlogPoct	20.6	21.3	22.4	24.9	21.0	21.3	22.0	20.9	21.4	22.1	22.6	8.0–35.0
QPlogPw	12.4	12.0	12.9	15.0	12.2	12.6	11.8	13.6	13.6	14.5	14.3	4.0–45.0
QPlogPo/w	2.5	3.4	2.8	2.8	3.0	2.5	3.3	1.8	1.8	1.9	2.5	-2.0–6.5
QPlogS	-4.5	-5.8	-5.3	-5.3	-5.4	-4.6	-5.7	-4.7	-4.5	-4.4	-5.2	-6.5–0.5
CIQPlogS	-5.1	-6.4	-5.5	-6.3	-6.7	-5.3	-5.9	-5.5	-5.5	-5.1	-6.7	-6.5–0.5
QPlogHERG	-5.9	-5.7	-5.9	-6.2	-5.8	-5.6	-5.8	-5.8	-5.8	-5.7	-5.7	*
QPPCaco (nm/sec)	258	258	226	134	258	258	258	26	38	100	100	**
QPlogBB	-1.4	-1.1	-1.6	-1.8	-1.2	-1.4	-1.5	-2.6	-2.4	-1.9	-1.8	-3.0–1.2
QPPMDCK (nm/sec)	234	1144	202	114	620	234	232	20	28	82	218	**
QPlogKp	-3.1	-3.4	-3.4	-3.4	-3.3	-3.3	-3.3	-5.2	-4.8	-3.9	-4.1	Kp in cm/hr
IP (ev)	8.6	8.7	8.6	8.5	8.6	8.6	8.6	8.8	8.8	8.7	8.7	7.9–10.5
EA (eV)	1.9	2.1	1.9	1.8	2.0	1.9	1.8	2.7	2.9	2.0	2.2	-0.9–1.7
#metab	2	2	3	3	2	3	3	3	3	3	3	1–8
QPlogKhsa	-0.1	0.1	0.0	0.1	0.0	-0.2	0.2	-0.2	-0.2	-0.2	-0.1	-1.5–1.5
Human Oral Absorp.	3	3	3	3	3	3	3	2	2	3	3	-
Percent Human Oral Absorp.	85	90	73	69	88	72	90	50	53	61	64	***
PSA	118	118	124	135	118	128	118	167	165	140	140	7–200
RuleOfFive	0	0	1	1	0	1	0	1	1	1	1	Maximum is 4
RuleOfThree	0	1	0	0	0	0	1	0	0	0	0	Maximum is 3
Jm	0.0	0.0	0.0	0.0	0.0	0.0	0.0	0.0	0.0	0.0	0.0	**-**

*(concern below -5)

** <25 = poor and >500 = great

*** <25% = poor and >80% = high.

All ADME/T parameters obtained above are explained. In the light of these explanations, it is seen that the numerical values of ADME/T parameters of these molecules are within the limit ranges. In particular, the numeric values of the RuleOfFive and RuleOfThree parameters are generally zero, which is the lower limit. Using this information, it has been seen that these molecules are suitable as drugs for human metabolism.

## Conclusion

Azomethine derivatives of acefylline tethered 1,2,4-triazole derivatives (**7a-k)** were afforded in good yield and further their anticancer, thrombolytic, and hemolytic activities were evaluatedare reported in this paper. The anticancer activity of all the compounds of synthesized series was evaluated against liver cancer cell line (Hep G2). Many compounds exhibited better anticancer activity, compound **7d** with the least cell viability value (11.71 ± 0.39%) using 100μg/μl concentration of compound was be the most active anticancer agent. The activities of the studied derivatives against liver cancer were compared, and it was seen that molecule **7d** had higher activity than other molecules. After molecular modeling ADME/T analysis was also performed to assess the interaction of synthesized series of molecules on human metabolism. It was seen that the calculated parameters met the conditions for being a drug for all molecules.

The clot lysis evaluations were moderate presenting **7k** (52.1% clot lysis) and in the hemolysis assay, nearly all molecules exhibited low toxicity against human RBCs. Compound **7g** with 0.26% hemolysis was found to be the least toxic compound. Recent studies show that further modifications on acefylline-derived azomethine-triazole hybrids can lead the advanced anti-cancer agents.

## Supporting information

S1 File (DOC)
