# Supplementary material for: Repositioning of acefylline as anti-cancer drug: Synthesis, anticancer and computational studies of azomethines derived from acefylline tethered 4-amino-3-mercapto-1,2,4-triazole
Source: PLoS One. 2022 Dec 15;17(12):e0278027. doi: 10.1371/journal.pone.0278027 (PMC9754256; doi:10.1371/journal.pone.0278027)
Supplement: S1 File — (DOC) [file pone.0278027.s001.doc]

**Repositioning of Acefylline as Anti-cancer Drug: Synthesis, Anticancer and Computational Studies of Azomethines Derived from Acefylline Tethered 4-Amino-3-Mercapto-1,2,4-Triazole**

Irum Shahzadi^1^, Ameer Fawad Zahoor*^,1^, Burak Tüzün^2^, Asim Mansha^1^, Muhammad Naveed Anjum^3^, Azhar Rasul^4^, Ali Irfan^1^, Katarzyna Kotwica-Mojzych^5^ and Mariusz Mojzych^6,*^

^1^Department of Chemistry, Government College University Faisalabad, 38000-Faisalabad, Pakistan. [irumshahzadi216@gmail.com](mailto:irumshahzadi216@gmail.com), [mansha.asim@gmail.com](mailto:mansha.asim@gmail.com), [raialiirfan@gmail.com](mailto:raialiirfan@gmail.com)

^2^ Plant and Animal Production Department, Technical Sciences Vocational School of Sivas, Sivas Cumhuriyet University, 58140-Sivas, Turkey. [theburaktuzun@yahoo.com](mailto:theburaktuzun@yahoo.com)

^3^ Department of Applied Chemistry, Government College University Faisalabad, 38000-Faisalabad, Pakistan. [anjumccj@hotmail.com](mailto:anjumccj@hotmail.com)

^4^ Department of Zoology, Government College University Faisalabad, 38000-Faisalabad, Pakistan. [drazharrasul@gmail.com](mailto:drazharrasul@gmail.com)

^5^ Laboratory of Experimental Cytology, Medical University of Lublin, Radziwiłłowska 11, 20-080 Lublin, Poland; [katarzynakotwicamojzych@umlub.pl](mailto:katarzynakotwicamojzych@umlub.pl)

6 Department of Chemistry, Siedlce University of Natural Sciences and Humanities, 3-go Maja 54, 08-110 Siedlce, Poland

* Corresponding author: Ameer Fawad Zahoor: [fawad.zahoor@gcuf.edu.pk](mailto:fawad.zahoor@gcuf.edu.pk); Mariusz Mojzych: [mariusz.mojzych@uph.edu.pl](mailto:mariusz.mojzych@uph.edu.pl);

^1^H and ^13^C NMR spectra of compound **6** and **7a**-**k**

^13^C NMR spectrum of compound **6.**

S
